# Fungi Isolated from Maize (*Zea mays* L.) Grains and Production of Associated Enzyme Activities

**DOI:** 10.3390/ijms160715328

**Published:** 2015-07-07

**Authors:** Camila Agnes Lumi Abe, Carla Bertechini Faria, Fausto Fernandes de Castro, Sandra Regina de Souza, Fabiane Cristina dos Santos, Cleiltan Novais da Silva, Dauri José Tessmann, Ione Parra Barbosa-Tessmann

**Affiliations:** 1 Department of Biochemistry, State University of Maringá, Av. Colombo, 5790, Maringá 87020-900, Brazil; E-Mails: camilaagneslumiabe@gmail.com (C.A.L.A.); cbertechini.faria@gmail.com (C.B.F.); fausto_fernandes25@hotmail.com (F.F.C.); sandrasouzaa@yahoo.com.br (S.R.S.); fabianecristina.santos@gmail.com (F.C.S.); 2 Department of Agronomy, State University of Maringá, Av. Colombo, 5790, Maringá 87020-900, Brazil; E-Mails: cleiltan@gmail.com (C.N.S.); djtessmann@uem.br (D.J.T.)

**Keywords:** amylase, cellulase, protease, lipase, fungi, corn

## Abstract

Filamentous fungi produce a great variety of enzymes, and research on their biotechnological potential has recently intensified. The objective of this work was to identify, at the species level, using DNA barcoding, 46 fungal isolates obtained from maize grains with rot symptoms. We also analyzed the production of extracellular amylases, cellulases, proteases and lipases of 33 of those fungal isolates. The enzymatic activities were evaluated by the formation of a clear halo or a white precipitate around the colonies in defined substrate media. The found fungi belong to the genera *Talaromyces*, *Stenocarpella*, *Penicillium*, *Phlebiopsis*, *Cladosporium*, *Hyphopichia*, *Epicoccum*, *Trichoderma*, *Aspergillus*, *Irpex*, *Fusarium*, *Microdochium*, *Mucor* and *Sarocladium*. In the genus *Fusarium*, the species *Fusarium verticillioides* was predominant and this genus presented the highest diversity, followed by the genera *Aspergillus*. The best genera for lipase production were *Cladosporium* and *Penicillium*; while *Cladosporium*, *Aspergillus* and *Penicillium* were best for cellulase activity; *Hyphopichia*, *Aspergillus* and *Irpex* for amylase activity; and *Cladosporium* and *Sarocladium* for proteases activity. In conclusion, a collection of fungi from maize seeds presenting rotten symptoms were obtained, among which exist important producers of hydrolases.

## 1. Introduction

The industrial processes that use enzymes are important because they are simple and have low cost, low environmental impact and low energy requirements. Unlike chemical catalysts in industrial processes, enzymes are biodegradable, act under mild conditions of pH and temperature, reduce the reaction time, save energy and do not cause deterioration of industrial materials [1]. The global market for industrial enzymes is expected to reach more than 4 billion dollars by 2015 [2]. Hydrolases is the most commonly employed class of enzymes, and includes amylases, proteases, cellulases and lipases [1,2]. The industrial enzymes market prefers microbial enzymes because they are more stable than enzymes from plants and animals [3]. In particular, fungi are preferred for enzyme production, since they grow easily in a diversity of substrates and the large scale production of enzyme is a relatively easy process in biotechnological industries. Because of the large diversity of organisms present in nature, much research is currently being aimed at the discovery of new producer microorganisms and enzymes [3].

Amylases account for 25% of global enzyme production [2]. Endoamylases (e.g., α-amylases) and exoamylases (e.g., glucoamylases and β-amylases) are the most common amylases [4]. Glucoamylases and α-amylases have been identified in several microorganisms [5,6]. α-Amylase may be derived from several bacteria, yeasts and fungi, but the bacterial enzyme is generally preferred due to several characteristic advantages that it offers [5]. Filamentous fungi apparently constitute the major source of glucoamylases among all microbes [6]. The fungi synthesizing glucoamylases active at higher temperatures include *Aspergillus* sp., *Mucor* sp., *Neurospora crassa*, *Rhizopus* sp. and *Arthrobotrys amerospora* [4,6]. β-amylase, which is generally of plant origin, has also been reported from a few microbial sources, mainly from bacteria but also from some fungal strains (e.g., *Rhizopus* sp.) [4]. Amylases are of great biotechnological importance, being used in the following industries: textile, brewery, bakery, cereal for infants, animal feeding, distilled beverage, starch liquefaction and saccharification, chemistry, pharmaceutical and bioethanol production [4,7].

There are three main classes of cellulases: endoglucanases, exoglucanases and β-glucosidases [8,9,10]. Fungal cellulolytic enzymes are advantageous because they are secreted as an enzyme complex that works in a synergistic manner, and their production is a relatively easy and inexpensive process [10]. Currently, most commercial cellulases (including β-glucosidase) are produced by *Trichoderma* and *Aspergillus* species [11]. Cellulases are sold in a great volume and are applied in the following industries: food, animal feeding, starch processing, ethanol production to be used as fuel, fruit juice and vegetal pulp extraction, paper, brewery and wine, textile and laundry, as well as in agriculture and research [8,9,10].

Proteases are classified according the pH in which they are active (acid, neutral and alkaline proteases), according on how they act on substrate (endo or exopeptidases, which are subdivided in amino or carboxipeptidases), according to the amino acid or other element present in their active site (serine, aspartic, cysteine, threonine or metalo proteases) and according to their amino acid sequences and evolutionary affinities. Most commercial proteases, mainly neutral and alkaline, are produced by bacteria belonging to the genus *Bacillus* [12]. Fungus such as *Aspergillus oryzae* produce acid, neutral and alkaline proteases. Regarding filamentous fungal exopeptidases, there have been reports of aminopeptidases production by *A. oryzae* and of serine carboxypeptidases from *Penicillium* spp. and *Aspergillus* spp. [13]. Considering filamentous fungal endopeptidases, there have been reports of alkaline serine proteases production by *Blakeslea trispora*, *Conidiobolus* spp., *Aspergillus* sp. and *Neurospora* spp. and of aspartic proteases by *Aspergillus*, *Penicillium*, *Rhizopus*, *Neurospora*, *Endothia* and *Mucor* spp. [13]. Proteases constitute the largest product segment in the global industrial enzymes market [1]. However, until today, the largest share of the enzyme market has been held by detergent alkaline proteases [12]. In addition to their use in detergents, proteases can also be used in food industries, leather treatment, in several bioremediation processes and in the pharmaceutical industry for preparation of medicines such as ointments for debridement of wounds.

Esterases hydrolyze short chain fatty acids esters and lipases hydrolyze long chain fatty acids esters, mainly triacylglycerol [14,15,16]. Numerous species of bacteria, yeasts and molds produce lipases [17]. Although a large number of microbial strains have been used for production of these enzymes, *Candida* sp., *Pseudomonas* sp. and *Rhizopus* sp. are the most important sources. *Candida rugosa* is considered the most frequently used organism for lipase synthesis [17]. Among ascomycetes, lipase is mainly produced by *Geotrichum candidum*, *Aspergillus* spp., *Penicillium* spp. and *Fusarium* spp. [17]. After proteases and amylases, lipases are considered the third most important enzyme group in terms of volume of sales [2]. Lipases are used in the detergent, paper and food industries, and in effluent treatment. In organic interfaces, lipases also perform esters synthesis [18]. An important ester of fatty acids with ethanol or methanol is biodiesel, an alternative fuel. The biodiesel industrial production uses lipases, but not extensively, although the enzyme process presents several advantages over the chemical process [19].

Fungi used for biotechnological purposes in the search for enzyme production can be obtained from culture collections and/or from natural sources that are rich in the enzyme substrates. Maize (*Zea mays* L.) is a commercial plant that originates from the Americas, but that has been cultivated in several hot and temperate regions around the world. The economic importance of maize is characterized by its high nutritional value; it is rich in starch, protein, triacylglycerol and fibers. Faria *et al.* [20] have isolated fungi from maize grains presenting rotten symptoms. In view of the rot symptoms, it was assumed that these isolated fungi might produce hydrolases for degrading maize grain components. 

## 2. Results and Discussion 

### 2.1. Species Diversity

After searching sequence databanks for DNA homologous to the obtained 5.8S-ITS rDNA and Translation Elongation Factor 1α gene (*TEF1α*) sequences, a range of identity from 90% to 100% was determined (Table 1). A great diversity was found within filamentous fungi. Sixteen fungal species were identified in total (Table 1). In the genus *Fusarium*, the species *Fusarium verticillioides* was the most frequently found and this genus presented the highest diversity. The genera *Aspergillus* was the second most diverse. The isolate PG 1-1 was previously morphologically identified as *Fusarium circinatum* [20], but in this work the rDNA sequencing found this is actually an isolate of *Sarocladium zeae*. Using the *TEF1α* sequences, three isolates of *F. verticillioides* and two isolates of *F. subglutinans*, which were previously identified in PCR reactions with specific primers [20], had their identification confirmed by BLAST analysis in the FUSARIUM-ID platform [21] and GenBank.

**Table 1:** Fungal isolates and molecular identification.

Genera and Species	Isolate	Geographic Origin (City, State)	GenBank Accession Number	GenBank or Fusarium-ID (FD) Accession Number of Similar Sequences (Percentage of Identity)
*Aspergillus flavus*	PG 24	Ponta Grossa, PR	KP691048	KP036603.1 (100%), KM486551.1 (100%)
*Aspergillus* sp.	BAN 12	Bandeirantes, PR	-	#
*Aspergillus* sp.	MGA 20	Maringá, PR	-	#
*Aspergillus wentii*	PG 18	Ponta Grossa, PR	KP691043	KM409566.1 (100%), AY373884.1 (100%)
*Cladosporium cladosporioides*	CMA 15	Clementina, SP	KP691040	KJ589558.1 (100%), KF986546.1 (100%)
*C. cladosporioides*	CMA 07	Clementina, SP	KP691034	KJ589558.1 (100%), JX406506.1 (100%)
*Curvularia* sp.	MGI 04	Mandaguari, PR	-	#
*Epicoccum nigrum*	PG 16	Ponta Grossa, PR	KP691041	JX844158.1 (100%), KJ934366.1 (99%)
*E. nigrum*	PG 23	Ponta Grossa, PR	KP691047	KF990155.1 (99%), KC005662.1 (99%)
*E. nigrum*	CMA 14	Clementina, SP	KP691039	JX844158.1 (100%), KJ934366.1 (99%)
*Epicoccum sorghinum*	CO 11	Cruzeiro do Oeste, PR	KP691037	KM111488.1 (100%), KP050561.1 (99%)
*Fusarium incarnatum-equiseti* *	RV 18-1	Rio Verde, GO	KP336404	KF962948.1 (97%), FD_01623 (97.5%)
*F. incarnatum-equiseti* *	CMA 1-2	Clementina, SP	KP336405	KF962948.1 (97%), FD_01623 (97.5%)
*F. incarnatum-equiseti* *	CMA 5-1	Clementina, SP	KP336406	KF962948.1 (97%), FD_01623 (97.5%)
*Fusarium subglutinans* *	RV 23-2	Rio Verde, GO	KP336408	JX867945.1 (100%), KC964122.1 (98%)
*F. subglutinans* *	PG 1-2	Ponta Grossa, PR	KP336409	KC964122.1 (99%), JF270302.1 (99%),
*Fusarium verticillioides*	CMA 3-1	Clementina, SP	KP691051	KJ125822.1 (100%), KJ125764.1 (100%)
*F. verticillioides*	RV 8-1	Rio Verde, GO	KP691052	KJ125822.1 (100%), KJ125764.1 (100%)
*F. verticillioides*	BAN 4-2	Bandeirantes, PR	KP691053	KJ125822.1 (100%), KJ125764.1 (100%)
*F. verticillioides*	MGI 1-1	Mandaguari, PR	KP691054	KJ125822.1 (100%), KJ125764.1 (100%)
*F. verticillioides*	BAN 2-2	Bandeirantes, PR	KP691055	KJ125822.1 (100%), KJ125764.1 (100%)
*F. verticillioides*	MGA 5-1	Maringá, PR	KP691056	KJ125822.1 (100%), KJ125764.1 (100%)
*F. verticillioides*	RV 12-2	Rio Verde, GO	KP691057	KJ125822.1 (100%), KJ125764.1 (100%)
*F. verticillioides*	MGA 2-2	Maringá, PR	KP691058	KJ125822.1 (100%), KJ125764.1 (100%)
*F. verticillioides*	CMA 2-1	Clementina, SP	KP691059	KJ125822.1 (100%), KJ125764.1 (100%)
*F. verticillioides*	CPÃ 1-1	Camapuã, MS	KP691060	KJ125822.1 (100%), KJ125764.1 (100%)
*F. verticillioides* *	MGI 3-2	Mandaguari, PR	KP336410	KF715263.1 (100%), KC599244.1 (99%)
*F. verticillioides* *	RV 12-2	Rio Verde, GO	KP336411	KF715263.1 (100%), KC599244.1 (99%)
*F. verticillioides* *	PG 4-1	Ponta Grossa, PR	KP336412	KF715263.1 (100%), KC599244.1 (99%)
*Fusarium andiyazi* *	RV 27-1	Rio Verde, GO	KP336407	JX974611.1 (99%), KC954401.1 (98%)
*Fusarium graminearum*	MGI 21	Mandaguari, PR	KP691045	KM513614.1 (100%), KF624778.1 (100%)
*Fusarium nygamai* *	RV 27-2	Rio Verde, GO	KP336403	JF740790.1 (95%), HM243236.1 (95%)
*Hyphopichia burtonii*	CMA 09	Clementina, SP	KP691036	KP132302.1 (99%), EU714323.1 (98%)
*Irpex lacteus*	CMA 19	Clementina, SP	KP691044	LN714557.1 (100%), KJ831879.1 (100%)
*Microdochium nivale*	PG 22	Ponta Grossa, PR	KP691046	JX280606.1 (98%), EF187912.1 (92%),
*Mucor fragilis*	CMA 25	Clementina, SP	KP691049	JQ972063.1 (99%), FN650655.1 (99%)
*Mucor* sp.	CO 10	Cruzeiro do Oeste, PR	-	#
*Penicillium digitatum*	RV 06	Rio Verde, GO	KP691033	AY373910.1 (100%), KJ834506.1 (100%)
*Penicillium* sp.	MGI 01	Mandaguari, PR	-	#
*Phlebiopsis gigantea*	CMA 08	Clementina, SP	KP691035	JQ781838.1 (98%), FJ791151.1 (98%)
*Sarocladium zeae*	PG 1-1	Ponta Grossa, PR	KP691050	KP132614.1 (100%), KJ188657.1 (98%)
*Stenocarpella maydis*	MGI 03	Mandaguari, PR	KP691031	KM030331.1 (100%), KC311732.1 (100%)
*Talaromyces purpureogenus*	MGI 05	Mandaguari, PR	KP691032	JX157861.1 (99%), JQ422620.1 (99%)
*T. purpureogenus*	PG 17	Ponta Grossa, PR	KP691042	JX157861.1 (99%), JQ422620.1 (99%)
*T. purpureogenus*	MGI 02	Mandaguari, PR	KP691030	JX157861.1 (99%), JQ422620.1 (99%)
*Trichoderma harzianum*	CO 13	Cruzeiro do Oeste, PR	KP691038	KP050785.1 (100%), KF624792.1 (100%)

* *TEF1α* sequence; # Isolates MGI 1, MGI 04, CO 10, Ban 12 and MGA 20 were only morphologically identified. GO—Goiás; MS—Mato Grosso do Sul; PR—Paraná; SP—São Paulo.

**Figure 1:**
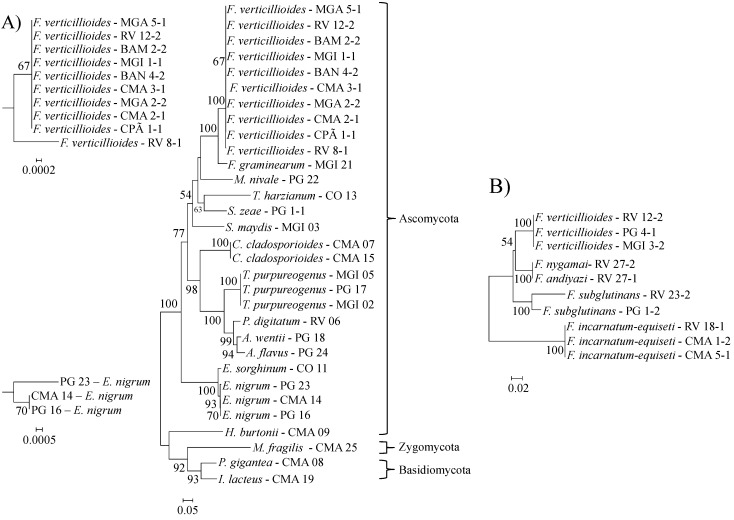
The evolutionary history of the rDNA (**A**) and *TEF1α* (**B**) sequences. The optimal trees with the sum of branch length = 2.85730321 for rDNA and 0.36769626 for *TEF1α* are shown. The percentage of replicate trees in which the associated taxa clustered together in the bootstrap test (1000 replicates) is shown next to the branches [26]. Bootstrap values lower than 50% are not shown. The trees are drawn to scale, with branch lengths in the same units as those of the evolutionary distances used to infer the phylogenetic tree. The analysis involved 31 nucleotide sequences for rDNA and 10 sequences for *TEF1α*. All ambiguous positions were removed for each sequence pair. There were a total of 491 positions for rDNA and 645 for *TEF1α* in the final dataset. In (**A**) the *F. verticillioides* and *E. nigrum* branches are magnified. Evolutionary analyses were conducted in MEGA6 [27].

Most (91.31%) of our isolates were Ascomycota, but Zygomycota (4.34%) and Basidiomycota (4.34%) were also found (Figure 1A). Most of the obtained fungus genera are frequently found in maize (e.g., *Aspergillus*, *Cladosporium*, *Curvularia*, *Fusarium*, *Mucor*, *Penicillium* and *Trichoderma*) [22,23]. Most of the existent literature [22,23] describes the occurrence of filamentous fungi from the phylum Ascomycota, because those studies were done by morphological identification. Ascomycetes can form spores under laboratory conditions and the morphology of these spores can be used in the fungus identification. In addition to common ascomycetes, here we describe, at the species level, the occurrence of zygomycetes and basidiomycetes in maize seeds. The identification of those fungi was only possible because of the particular molecular methodology applied here. The data also reveals that there is diversity among the isolates of the same species as *F. verticillioides* and *E. nigrum* (Figure 1A). There was one isolate of *F. verticillioides* that differed from the others. Molecular diversity among *F. verticillioides* isolates associated with maize seeds in Brazil have been previously described [24]. The diversity among *E. nigrum* DNA sequences and other profiles, including enzyme secretion, has also been described [25]. The authors of this previous work have divided *E. nigrum* into two groups and even proposed that one of those groups is a new species. The partial sequencing of the *TEF1α* gene of *F. subglutinans* has also indicated genetic variability among the two recognized isolates (Figure 1B). The occurrence of *F. subglutinans* in maize in Brazil is rare and there have been no reports about its diversity. The *TEF1α* sequences of isolates RV 27-1 and RV 27-2 did not show any difference. The RV 27-2 isolate was previously [20] identified as *F. nygamai* using specific primers and PCR, which did not recognize the RV 27-1 isolate. It is very difficult to differentiate among species of the *Gibberella fujikuroi* species complex as *F. verticillioides*, *F. subglutinans*, *F. nygamai* and *F. andiyazi*, and the identity of the RV 27-1 isolate, which was classified as *F. andiyazi*, requires further investigation.

### 2.2. Extracellular Enzyme Activities

**Figure 2:**
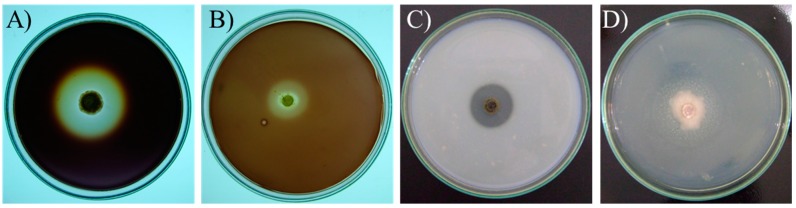
Petri dishes with defined substrate media for detection of the enzyme activities. (**A**) Amylolytic activity. Nutrient agar containing soluble starch. *A. wentii*-PG 18; (**B**) Cellulolytic activity. Medium containing microcrystalline cellulose. *P. digitatum*-RV 06; (**C**) Proteolytic activity. Medium containing milk casein. *C. cladosporioides*-CMA 15; (**D**) Lipolytic activity. Medium containing Tween 20 and CaCl_2_. *E. nigrum*-PG 16.

Extracellular amylase, cellulase, protease and lipase activities produced by the studied microorganisms were assessed in culture. The evaluation was performed by measuring the degradation halo of specific substrates (starch, microcrystalline cellulose, milk casein and tween 20, respectively) present in the solid media (Figure 2).

**Figure 3:**
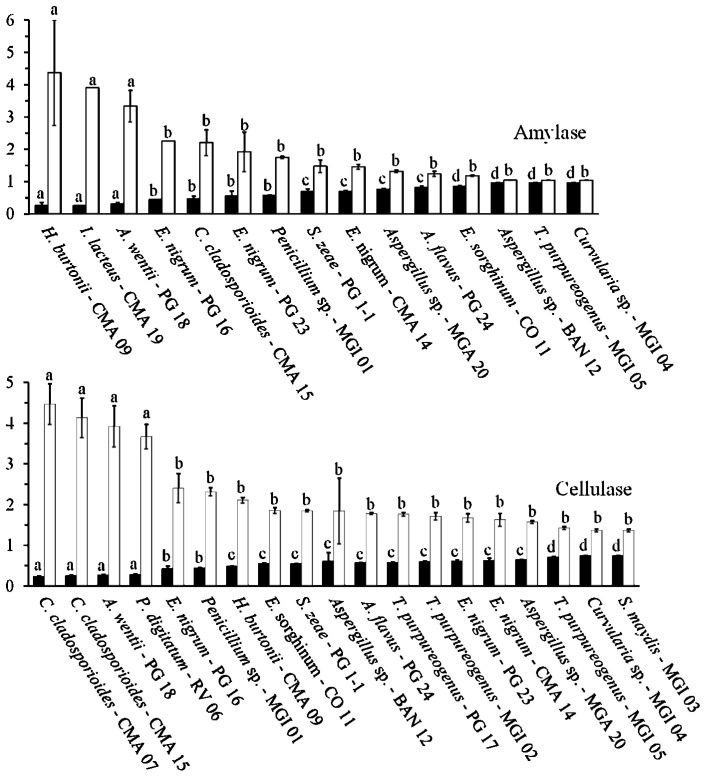

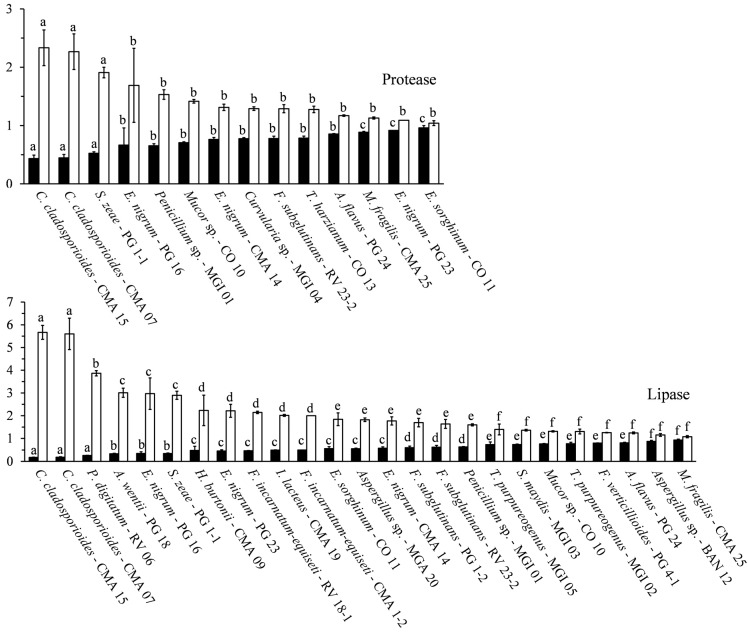
Enzyme activity analysis. The data are the average and standard deviation obtained in three repetitions. Enzyme activity was expressed as the Enzymatic Index (□) or as the 1-(C/H) index (■). Averages followed by the same letter are not significantly different according to the Scott-Knott test (α = 0.01). Averages are ordered following the Enzymatic Index.

All of the evaluated isolates were able to grow in the used media containing the specific substrates as the major carbon sources. However, after evaluation of the enzyme activities, only 28 (84.9%) of the 33 tested fungal isolates produced at least one type of the studied enzyme activities, with the used methodology. In total, 15 (45.5%) of the isolates exhibited amylase enzyme activity, 19 (57.6%) cellulase activity, 14 (42.4%) protease activity and 25 (75.8%) lipase activity (Table 2, Figure 3). However, among the isolates that have produced the enzymes, only 12 fungal isolates (36.4%) were considered as good producers, according the evaluation of the EI ≥ 2.0, as established as standard.

**Table 2:** Enzymatic Index of hydrolytic enzymes production by fungi isolated from maize with rot symptoms.

Fungal Strain	Strain	Amylase *	Cellulase *	Protease *	Lipase *
*A. flavus*	PG 24	1.24 ± 0.08 ^b^	1.78 ± 0.03 ^b^	1.17 ± 0.01 ^b^	1.24 ± 0.04 ^f^
*A. wentii*	PG 18	3.33 ± 0.49 ^a^	3.92 ± 0.51 ^a^	-	3.01 ± 0.20 ^c^
*Aspergillus* sp.	BAN 12	1.04 ± 0.00 ^b^	1.84 ± 0.81 ^b^	-	1.15 ± 0.06 ^f^
*Aspergillus* sp.	MGA 20	1.31 ± 0.04 ^b^	1.57 ± 0.04 ^b^	-	1.82 ± 0.08 ^e^
*C. cladosporioides*	CMA 07	-	4.47 ± 0.50 ^a^	2.27 ± 0.31 ^a^	5.60 ± 0.69 ^a^
*C. cladosporioides*	CMA 15	2.20 ± 0.40 ^b^	4.13 ± 0.50 ^a^	2.33 ± 0.31 ^a^	5.67 ± 0.31 ^a^
*Curvularia* sp.	MGI 04	1.04 ± 0.00 ^b^	1.36 ± 0.04 ^b^	1.29 ± 0.03 ^b^	-
*E. nigrum*	CMA 14	1.46 ± 0.08 ^b^	1.62 ± 0.16 ^b^	1.31 ± 0.06 ^b^	1.77 ± 0.18 ^e^
*E. nigrum*	PG 16	2.25 ± 0.00 ^b^	2.40 ± 0.36 ^b^	1.69 ± 0.63 ^b^	2.97 ± 0.69 ^c^
*E. nigrum*	PG 23	1.92 ± 0.62 ^b^	1.67 ± 0.11 ^b^	1.09 ± 0.00 ^b^	2.21 ± 0.29 ^d^
*E. sorghinum*	CO 11	1.18 ± 0.03 ^b^	1.85 ± 0.08 ^b^	1.04 ± 0.04 ^b^	1.84 ± 0.28 ^e^
*F. andiyazi*	RV 27-1	-	-	-	-
*F. graminearum*	MGI 21	-	-	-	-
*F. incarnatum-equiseti*	RV 18-1	-	-	-	2.00 ± 0.00 ^d^
*F. incarnatum-equiseti*	CMA 1-2	-	-	-	2.14 ± 0.05 ^d^
*F. nygamai*	RV 27-2	-	-	-	-
*F. subglutinans*	RV 23-2	-	-	-	1.70 ± 0.19 ^e^
*F. subglutinans*	PG 1-2	-	-	1.29 ± 0.07 ^b^	1.64 ± 0.20 ^e^
*F. verticillioides*	MGI 3-2	-	-	-	-
*F. verticillioides*	RV 12-2	-	-	-	-
*F. verticillioides*	PG 4-1	-	-	-	1.26 ± 0.01 ^f^
*H. burtonii*	CMA 09	4.37 ± 1.64 ^a^	2.10 ± 0.07 ^b^	-	2.23 ± 0.67 ^d^
*I. lacteus*	CMA 19	3.90 ± 0.00 ^a^	-	-	2.01 ± 0.04 ^d^
*M. fragilis*	CMA 25	-	-	1.13 ± 0.02 ^b^	1.08 ± 0.05 ^f^
*Mucor* sp.	CO 10	-	-	1.42 ± 0.03 ^b^	1.31 ± 0.03 ^f^
*P. digitatum*	RV 06	-	3.67 ± 0.31 ^a^	-	3.87 ± 0.12 ^b^
*Penicillium* sp.	MGI 01	1.74 ± 0.04 ^b^	2.31 ± 0.10 ^b^	1.53 ± 0.08 ^b^	1.60 ± 0.05 ^e^
*S. maydis*	MGI 03	-	1.36 ± 0.04 ^b^	-	1.36 ± 0.04 ^f^
*S. zeae*	PG 1-1	1.47 ± 0.20 ^b^	1.84 ± 0.03 ^b^	1.91 ± 0.09 ^a^	2.90 ± 0.18 ^c^
*T. purpureogenus*	MGI 02	-	1.71 ± 0.09 ^b^	-	1.31 ± 0.11 ^f^
*T. harzianum*	CO 13	-	-	1.27 ± 0.06 ^b^	-
*T. purpureogenus*	MGI 05	1.04 ± 0.00 ^b^	1.42 ± 0.04 ^b^	-	1.40 ± 0.24 ^f^
*T.* *purpureogenus*	PG 17	-	1.76 ± 0.05 ^b^	-	-

* The data are the average and the standard deviation of the results obtained in three repetitions. Averages followed by the same letter (^a^, ^b^, ^c^, ^d^, ^e^ or ^f^) are not significantly different according to the Scott-Knott test (α = 0.01). Enzyme Index = R/r, where R is the degradation halo average diameter and r is the average colony diameter.

### 2.3. Amylolytic Activity

Fungi can use starch as an energy source for growth and sporulation. However, amylase production by filamentous fungi varies across genera and species [4,5,6,7]. In this study, we found that the following fungal isolates were among the best amylase producers: *H. burtonii*, *Irpex lacteus*, *A. wentii*, *E. nigrum* and one isolate of *C. cladosporioides* (Table 2, Figure 3), with *H. burtonii* being the best producer of the amylolytic activity (IE = 4.37). The production of amylase in liquid culture by *H. burtonii* [28], *I. lacteus* [29] and *A. wentii* [30] has been previously described. The production in solid media of amylase by *E. nigrum* [25] and *C. cladosporioides* [31] has also been described. The amylase enzymes produced by *H. burtonii* and *I. lacteus* are α-amylases with low optimum temperatures: 40 and 50 °C, respectively [28,30]. The amylases produced by *A. wentii*, *E. nigrum* and *C. cladosporioides* have yet to be characterized. Further study of the enzymes from these species may result in the discovery of new enzymes with novel properties.

### 2.4. Cellulolytic Activity

Among of the 19 fungi with cellulase activity, seven were considered as good producers (with an EI ≥ 2.0) (Table 2, Figure 3), namely the two isolates of *C. cladosporioides* (the highest producers), followed by the isolates of *A. wentii*, *P. digitatum*, *Penicillium* sp. and *H. burtonii*. The production of cellulase by *C. cladosporioides* [32], *A. wentii* [33] and *Penicillium* sp. [8,9,10,11] has been previously described. The enzymes from *C. cladosporioides* and *A. wentii* have been previously purified from liquid culture and characterized [32,33]. *C. cladosporioides* produces xylanase and cellulases active against carboxymethilcellulose (CMCase) and filter paper (FPase) [32] and A. *wentii* secretes a β-glucosidase [33]. However, the production of cellulase by *P. digitatum* and *H. burtonii* in solid medium was unknown. Interestingly, *I. lacteus*, a known producer of cellulase (commercial Driselase) [34,35], did not show cellulase activity in the screening test used here. Differences in protein expression among different isolates or differences in the produced enzyme ability in degrading the used substrate in the screening test could explain this result.

### 2.5. Proteolytic Activity

The only two isolates that had a proteolytic activity EI higher than 2.0 were the two isolates of *C. cladosporioides* (Table 2, Figure 3). However, the isolate of *S. zeae* PG 1-1 had a production profile that was not statistically different from the two isolates of *C. cladosporioides*. In addition, the isolate of *E. nigrum* PG 16 was the fourth highest producer. The hydrolysis of casein in solid medium by *C. cladosporioides* was also seen by other authors [36] but the produced protease has not been purified or characterized. The production of protease activity in solid medium by *S. zeae* and *E. nigrum* is a novel finding. Basiomycetes can produce a diversity of proteases [37] and the presence of proteases in the secretome of *I. lacteus* [38] has been evidenced. However, no extracellular proteolytic activity by the basidiomycetes *I. lacteus* was detected using our methodology. Differences in protein expression among different isolates or differences in the produced enzyme ability in degrading the substrate used in the screening test could explain this result.

### 2.6. Lipolytic Activity

Among of the 25 fungi with lipase activity, the two isolates of *C. cladosporioides* had the highest EI followed by *P. digitatum*, *A. wentii*, *S. zeae*, *E. nigrum*, *H. burtonii*, *I. lacteus* and *F. incarnatum-equiseti* (all with an EI ≥ 2.0) (Table 2, Figure 3). The production of lipases by *C. cladosporioides* [39], *A. wentii* [40], *E. nigrum* [25] and *H. burtonii* [18] has been previously reported. Nevertheless, the production of lipase by *P. digitatum*, *S. zeae*, *I. lacteus* and *F. incarnatum-equiseti* in solid medium was unknown. The enzyme from *C. cladosporium* was formerly characterized and it is more active on esters of medium-chain acids [39]. A liquid culture optimization has been performed to produce lipase from *A. wentii* [40], but its enzyme has not been characterized. The production of lipase by *E. nigrum* has not been optimized and the enzyme has also not been characterized. One of those enzymes might have a good profile on biodiesel synthesis.

### 2.7. Quantitative Analysis of Enzyme Production

**Figure 4:**
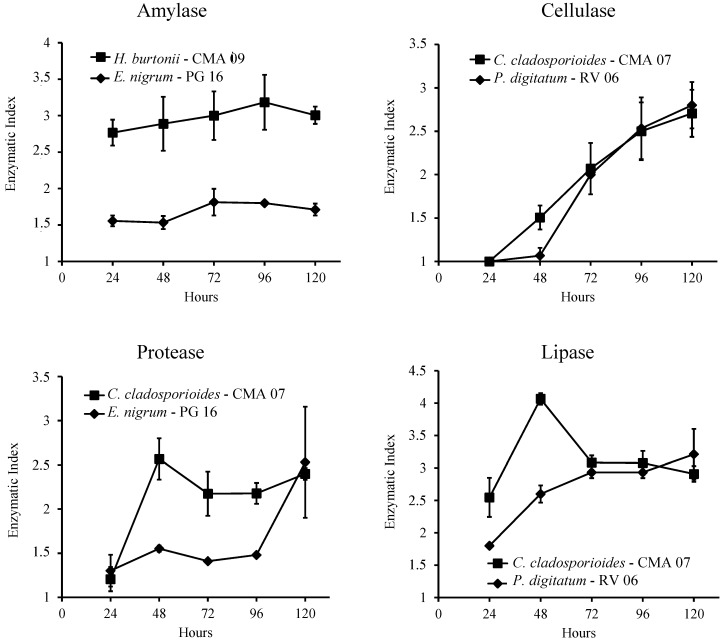
Time course of extracellular enzyme production by fungal producers on solid media containing the corresponding substrates. Enzymatic activity is expressed as Enzymatic Index (halo diameter/colony diameter). A known producer (■) and a novel producer (♦) were evaluated.

The results for the quantitative analysis of enzyme production are presented in Figure 4. A known producer (*H. burtonii* for amylase [28] and *C. cladosporioides* for cellulase, protease and lipase [32,36,39]) and a novel producer were evaluated for each enzyme. It is possible to see that the novel producers have a similar profile on enzyme production than the known producers.

## 3. Experimental Section

### 3.1. Fungal Isolates

A fungal collection obtained from maize grains showing signs of rot by Faria *et al.* [20] was used in this work (Table 1). To obtain this collection, Faria *et al.* [20] used corn spikes that were collected from January to October 2009, in fields in several maize producing regions of Brazil. The seeds of each spike were denominated as a lot and were kept in paper bags at 4 °C after insecticide treatment. A total of 57 maize seed lots were collected and analyzed. The isolates were obtained as follows. To obtain the *Fusarium* isolates, six maize seeds of each lot, in duplicate, were disinfected and inoculated in a 10 mm diameter dish containing Malaquite Green Agar (MGA) [41] supplemented with antibiotics. The seeds plated in the medium were incubated for 4 to 5 days at 25 °C, with a photoperiod of 12 h. After germination, mycelia and conidia of peach or violet colored colonies were transferred to Petri dishes containing Carnation Leaf-piece Agar (CLA) [42], which were incubated for a period of 7 days at 25 °C, with a photoperiod of 12 h. A well-colonized carnation leaf fragment in the CLA culture was used for monosporic isolation as described in Nelson *et al.* [43]. The isolates morphologic characteristics were analyzed in microculture performed in a 1 cm^3^ block of Spezieller Nährstoffarmer Agar (SNA) [42]. The isolates with cultural and morphological characteristics of *Fusarium* were cultivated for DNA extraction and molecular identification in PCR reactions with specific primers.

To obtain fungi other than *Fusarium* from maize seeds, six maize seeds of each lot, in triplicate, were disinfected and inoculated in a 10 mm diameter dish containing potato dextrose agar (PDA) [42], pH 4.5 with lactic acid [44], supplemented with antibiotics. The seeds plated in the medium were incubated for 5 to 7 days at 25 °C, with a photoperiod of 12 h. The fungi growing on the top and around the maize seeds with culture characteristics different from *G. fujikuroi* were submitted to monosporic isolation [43]. The morphological classification of the isolates was performed as described in Pitt and Hocking [45].

### 3.2. Molecular Identification of the Isolates

The species of the used isolates were identified by DNA extraction, amplification of the 5.8S-ITS rDNA region or a portion of the *TEF1α* gene, purification of the amplicon, sequencing, and comparison with other sequences deposited in data banks. 

For the DNA extractions, an approximately 1 cm^3^ fragment of a monosporic culture in inclined PDA was smashed and shaken in 5 mL of distilled water. Two milliliters of the obtained suspension was used as inoculum in 125 mL Erlenmeyer flasks containing 25 mL of potato dextrose medium. To prevent sporulation, the *Aspergillus* isolates were cultivated in 25 mL of liquid *A. flavus* and *Aspergillus* *parasiticus* (AFP) media containing 2% yeast extract; 1% bacteriological peptone; and 0.05% ammonium ferric citrate (*w*/*v*) [45]. These flasks were incubated for 5 days without shaking at 25 °C, with a photoperiod of 12 h. The mycelia were collected by filtration in sterile gauze. The mycelia was macerated in a mortar with liquid nitrogen, and transferred to microcentrifuge tubes. The DNA was extracted from the macerated mycelia using a protocol based in the method described by Koenig *et al.* [46], as follows. Approximately 700 μL of extraction buffer was added for each 300 μL of macerated material. The extraction buffer contained nuclear lyses buffer (0.2 M Tris, pH 7.5; 50 mM EDTA; and 2% (*w*/*v*) cetyltrimethylammonium bromide; pH 7.5), DNA isolation buffer (0.35 M sorbitol; 0.1 M Tris, pH 7.5; and 5 mM EDTA; pH 7.5), and 5% Sarkosyl, in the proportion of 1:1:0.4. The extraction buffer was combined right before use and was then added with 3.8 mg/mL of sodium bisulfite. The tubes were incubated in a dry bath at 65 °C for 60 min. After that, 500 μL of phenol-tris, pH 8.0, were added and the mixture was agitated by gentle inversion several times. The tubes were centrifuged at room temperature (12,000× *g*, 10 min), and the supernatant fraction was transferred to clean tubes. Next, 500 μL of a mixture of chloroform:isoamylic alcohol (24:1) were added to the supernatant and the mixture was agitated again by gentle inversion. The tubes were centrifuged at room temperature (12,000× *g*, 10 min), and the supernatant fraction was transferred to clean tubes. Samples were treated with 5 μL of RNAse A (20 mg/mL), for 30 min, at 37 °C, and next with 5 μL of proteinase K (20 mg/mL), for 30 min, at 56 °C. The DNA was then precipitated with an equal volume of isopropanol and was incubated at −20 °C overnight. The precipitated DNA was collected by centrifugation at room temperature (12,000× *g*, 10 min), and the DNA pellet was washed three times with cold 70% ethanol. The final DNA pellet was dried at room temperature and resuspended with 50 μL of TE buffer (10 mM Tris, pH 8.0; 1 mM EDTA). The whole process was carried out under sterile conditions. The DNA was quantified in a spectrophotometer at 260 nm and/or by fluorometry using the Qubit Quantitation Fluorometer and the Quant-it™ dsDNA HS Assay Kit (Life Technologies, Carlsbad, CA, USA). The DNA final concentration was adjusted to 100 ng/μL in TE buffer, and the DNA was kept frozen at −20 °C.

The amplification reactions were performed in a termocycler Techne TC-312 (Techne, Cambridge, UK) in PCR tubes containing 50 μL of the following reaction mixture: 1× enzyme buffer; 1.5 mM MgCl_2_, 1.5 U of Platinum *Taq* DNA polymerase (Life Technologies, Carlsbad, CA, USA); 0.2 mM of each dNTP (Life Technologies); 25 pmol of each primer (forward and reverse), and 400 ng of the DNA sample. The PCR reaction consisted of 25 cycles of 1 min and 30 s at 94 °C, 1 min and 30 s at 50 °C for the ITS4 and ITS5 primers [47] and 53 °C for the *TEF1α* primers [21], and 2 min at 72 °C. Prior to the cycles, the samples were heated for 5 min at 94 °C, and after the cycles the samples were incubated for 10 min at 72 °C and frozen at −20 °C until use. Negative controls (no DNA template) were used in each experiment to test for the presence of DNA contamination of reagents and reaction mixtures. Ten microliters of each PCR reaction were analyzed in a 1.5% agarose gel containing ethidium bromide (0.25 μg/mL). The PCR products were visualized and photographed under UV light. The rest of the PCR reaction was purified with the PureLink™ PCR purification kit (Life Technologies), or ExoSap-IT Kit (GE HealthCare, Milwaukee, WI, USA), or Wizard^®^ SV Gel and PCR Clean-Up System (Promega, Madison, WI, USA), and the amplified DNA was sequenced in the Center for the Human Genome Studies (CEGH) in the University of São Paulo (USP), Brazil.

Amplicons of approximately 500 and 700 bp were obtained from the 5.8S-ITS rDNA and *TEF1α* gene, respectively, and then sequenced. The primers used in sequencing were the same as those used for the amplification. The majority of the sequencing was done in both directions. After trimming at 5′ and 3′ extremities, the resulting sequences of 413 bp for the 5.8S-ITS rDNA and 623 bp for the *TEF1α* gene were compared with sequences deposited in data banks (GenBanK, Mycobank and Fusarium-ID) using MEGABLAST analysis. All obtained rDNA and *TEF1α* gene partial sequences were deposited in GenBank and the accession numbers are listed in Table 1. When the similarity degree was lower than 95%, macro morphological characteristics (e.g., colony color in BDA) and micro morphological characteristic (e.g., size and format of spores and presence of other micro structures) and analysis using traditional keys [45] and epidemiological data were used in the identification of the isolates.

To evaluate the phylogenetic distances of the fungi isolated from maize, the 5.8S-ITS rDNA (413 bp) and the *TEF1α* (623 bp) obtained sequences were aligned with ClustalW inside the MEGA 6.0 [27] program, and the alignment was used to construct a phylogenetic tree using the neighbor-joining method [48]. Values of 70% and above in the bootstrap test of phylogenetic accuracy have indicated reliable grouping among fungal isolates. The same grouping was also performed using other methods, such as maximum parsimony, minimum evolution and UPGMA with similar results. Pairwise deletion was used to remove gaps because a complete removal of the gaps could eliminate a large portion of phylogenetically meaningful sites. Bootstrap analyses were conducted to assess the confidence limits of the branching with 1000 heuristic replicates [26]. The evolutionary distances were computed using the maximum composite likelihood method [49] and are in the units of the number of base substitutions per site.

The DNA extracted of some isolates failed to produce a PCR product. Those isolates were classified only at the genera level, using the macro and micro characteristics and traditional keys [45] (Table 1). Two of the isolates died during the execution of this work: *Phlebiopsis gigantea* CMA 08 and *Microdochium nivale* PG 22. Those isolates and the isolate *F. incarnatum-equiseti* CMA 5-1 were not used in the enzyme activities analyses.

### 3.3. Detection of Extracellular Enzymatic Activity

The isolates were evaluated for their amylase, cellulase, protease and lipase activities in solid culture medium. The evaluation was based on the production of each enzyme in solid media as follows. Petri dishes containing solid culture medium with the specific substrate as the major carbon source were inoculated with the microorganisms and, after an incubation time, the results were evaluated by measuring the clear or the precipitated halo around the colonies, what indicated the production of the tested enzyme [50].

An approximately 0.5 cm^3^ fragment of a monosporic culture in inclined PDA or SNA [42] was transferred to 0.5 cm high BDA in 10 cm diameter Petri dishes. The dishes were incubated for 7 days at 25 °C with a photoperiod of 12 h. After growth, 0.5 cm diameter disks of the culture were cut with a sterile cork borer and transferred to the center of new 10 cm diameter Petri dish containing 0.5 cm high specific solid media with the inducer substrate as carbon source. Those dishes were incubated at 30 °C for 5 days, with a photoperiod of 12 h, before evaluation. Dishes containing fast growing fungi were incubated for only 2 days under the same conditions. The evaluation was performed in three dishes for each fungus and the results are expressed as the average of the triplicates.

The enzyme activity was determined by the method described by Hankin & Anagnostakis [51] with the enzymatic index (EI) expressed as EI = *R*/*r*, where *R* is the degradation halo diameter and *r* is the colony diameter. The fungal isolates presenting an EI higher or equal to 2.0 were considered as good producers of the enzyme activity under test in the used culture medium. The halo around the colony was measured in two diametrically opposed ways using a ruler in millimeters. Alternatively, enzyme activity was also expressed as 1-(C/H) where C is colony diameter and H is the diameter of the halo caused by substrate degradation [52]. In this analysis, the lower the index, the higher was the enzyme production.

The ability of the fungal isolates to degrade starch was used as a criterion to determine the production of amylases, using nutrient agar (3 g/L yeast extract, 5 g/L peptone, 15 g/L agar (*w*/*v*)) with 2 g/L of soluble starch [51]. After growth of the microorganisms, the dishes were treated with 5 mL of iodine reagent (1% KI; 0.5% I_2_ (*w*/*v*)) for the detection of the remaining substrate. The amylase activity was evaluated by the clear yellow halo around the colony of each isolate. 

Medium containing 7.0 g/L KH_2_PO_4_; 2.0 g/L K_2_HPO_4_; 0.1 g/L MgSO_4_·7H_2_O; 1.0 g/L (NH_4_)_2_SO_4_; 0.6 g/L yeast extract; and 10 g/L microcrystalline cellulose (Sigmacell, Sigma-Aldrich, St Louis, MO, USA); pH 5.5; and 15 g/L agar (*w*/*v*) was used for determination of cellulase activity production. After growth of the microorganisms for 2 or 5 days, as described above, and to accelerate the action of extracellular cellulases, which generally have optimum temperatures in this range, the dishes were incubated at 50 °C, for 16 h before evaluation [53]. After this period and to visualize the hydrolytic halo, the dishes were revealed with 5 mL of the iodine reagent [54] and distained by several washes in distilled water.

Protease producers were identified by the hydrolysis of casein in milk-agar [55], which was composed of 300 mL/L (*v*/*v*) of non-fat milk and 20 g/L agar (*w*/*v*). As the milk agar is opaque, the enzyme activity was evaluated by the presence of a degradation transparent halo around the colonies.

The lipolytic activity was determined by employing a culture media containing 10 g/L peptone; 5 g/L NaCl; 0.1 g/L CaCl_2_·2H_2_O; 17 g/L agar (*w*/*v*), and 10 mL/L of polioxiethilene sorbitan-monolaurate (Tween 20) (*v/v*) as lipid substrate [51,56]. The Tween 20 was autoclaved separately and added to the sterile media before pouring the media on the dishes. To improve the visualization of the precipitate, after culturing the microorganisms for 2 to 5 days, the dishes were incubated at 4 °C for 12 h, prior to evaluation. The lipolytic activity was evaluated by the presence of a white opaque precipitate around the colonies [51].

A quantitative assay of enzyme production in solid media was performed by measuring the hydrolysis halo over a period of 5 days. Two fungal strains were chosen for each enzyme. Protease and lipase halos were measured directly in three dishes of each fungus. Cellulase and amylase halos were measured in there dishes that were developed and discharged each day.

### 3.4. Statistical Analysis

The experiments were conducted as an entirely random delineation, with three repetitions. Statistical analyses were carried out by calculation of the means and standard deviations of the results. Where indicated, data was submitted to an ANOVA and compared using the Scott-Knott test (*p* < 0.01), using the SAS program (SAS Institute, Cary, NC, USA).

## 4. Conclusions

Here, we identified a great diversity of filamentous fungi in maize seeds presenting rot symptoms. A screening of some specific enzymes showed highly variable activity profiles, ranging from absent to strong. New producers (e.g., *P. digitatum* for cellulase and lipase and *E. nigrum* for protease) and producers whose enzymes were not fully characterized (e.g., *A. wentii* and *E. nigrum* for amylase and lipase) were found. A collection of hydrolase producing fungi was obtained. All detected enzyme activity might be used for improving technological processes. Future experiments of quantitative production and characterization of the produced enzymes will be carried on to evaluate their biotechnological potential.
